# A New Extraction System Based on Isopropyl Salicylate and Trioctylphosphine Oxide for Separating Alkali Metals

**DOI:** 10.3390/molecules27103051

**Published:** 2022-05-10

**Authors:** Aslan Yu. Tsivadze, Alexey A. Bezdomnikov, Vladimir E. Baulin, Liudmila I. Demina, Kirill P. Birin, Dmitriy V. Baulin, Yuliana I. Rogacheva

**Affiliations:** A.N. Frumkin Institute of Physical Chemistry and Electrochemistry of Russian Academy of Sciences, Leninsky pr. 31-4, 119071 Moscow, Russia; tsiv@phyche.ac.ru (A.Y.T.); mager1988@gmail.com (V.E.B.); ionh1961@rambler.ru (L.I.D.); kirill.birin@gmail.com (K.P.B.); badmitriy@gmail.com (D.V.B.); rogachevayuliana@gmail.com (Y.I.R.)

**Keywords:** lithium extraction, isopropyl salicylate, complex synthesis, DFT-calculation, FTIR spectroscopy, Raman spectroscopy, NMR spectroscopy

## Abstract

It was established that isopropyl salicylate can be used similarly to 1,3-diketones as a key component for a new efficient extraction system for selective separation of alkali metal cations. According to DFT modeling of complexes of isopropyl salicylate and 1,3-diketone with alkali metal cations (Li^+^, Na^+^, K^+^), six-membered metallacycles are formed whose stability decreases along the series Li > Na > K, which results in the observed enhanced affinity to lithium. The extraction ability of isopropyl salicylate is manifested in the presence of trioctylphosphine oxide (TOPO). The newly obtained complexes of isopropyl salicylate with alkali metal cations as well as their extracts in a mixture with TOPO are characterized by means of FT-IR, Raman, and NMR spectroscopy. The probable structure of the extracted lithium complex is presumed and the role of TOPO in the extraction process is investigated in detail. Extraction experiments showed extremely high separation coefficients for Li/Na and Li/K pairs in the extraction from a model multi-component solution.

## 1. Introduction

Lithium and Li-containing compounds are widely used in numerous areas of chemistry and aerospace, as well as medicine and nuclear energy [1,2,3,4]. The growing importance of electric vehicles and portable electronic devices requires greater raw material supplies for producing compounds used in high-efficiency lithium-ion batteries.

Due to the lack of mineral resources, it is required to consider the alternative ones, e.g., natural solutions (salt lake brines, geothermal water, and seawater) and industrial wastes, which contain most of the earth’s lithium stocks [3]. However, high hydration of the lithium cation and the presence of large excess of cations close to lithium in their physicochemical properties make the extraction of lithium from multi-component solutions a challenging task [5,6,7]. Therefore, the development of technologies for lithium extraction from natural and technological solutions is an important problem related to increasing production efficiency and wastes recycling.

Liquid-liquid extraction is one of the most commonly used technological processes of extraction of metal ions from multi-component solutions, mainly due to its high productivity and simplicity. Crown ethers, FeCl_3_-tributyl phosphate (TBP) and 1,3-diketones in mixtures with electron-donor additives are often considered as extractants for liquid-liquid lithium extraction. Crown ethers are not suitable for industrial application as a result of their low efficiency and high cost [8,9]. Systems based on the mixtures of FeCl_3_-TBP are commonly used in the extraction of lithium from brines with extremely high content of magnesium [10,11], but these systems are complex and sensitive to numerous parameters at the stages of extraction, washing, re-extraction, and regeneration [12]. Moreover, FeCl_3_-TBP-based systems cause corrosion of the equipment and consequently the products require additional treatment (neutralization, removal of admixtures, and sedimentation) [13,14].

**Scheme 1:**
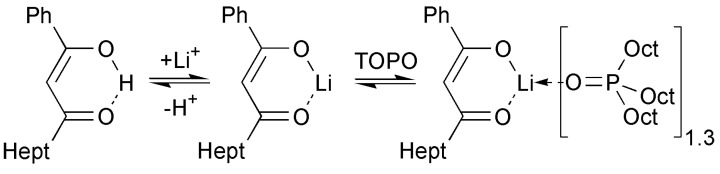
Mechanism of synergistic extraction of lithium with a mixture of phenyldecane-1,3-diketone and TOPO [25].

Mixtures of 1,3-diketones and neutral extractants, namely tributyl phosphate (TBP) and trioctylphosphine oxide (TOPO), quantitatively extract lithium from alkaline solutions containing high excess of Na^+^, K^+^ [15,16,17,18]. The degree of alkali cations extraction with such mixtures reasonably decreases in the series Li^+^ > Na^+^ > K^+^ with the increase of the ionic radii of alkali ions [15,19,20,21,22,23,24]. Enol form of 1,3-diketone chelate the lithium ion through the cation-exchange mechanism with the formation of a six-membered metallacycle and subsequent coordination with electron-donor molecules, such as TOPO [25] (Scheme 1). However, 1,3-diketones are not readily available, and their production and application can be accompanied by environmental risks. Thus, the search for new synthetically feasible and environmentally safe extractants for selective extraction of lithium is a promising challenge.

**Scheme 2:**
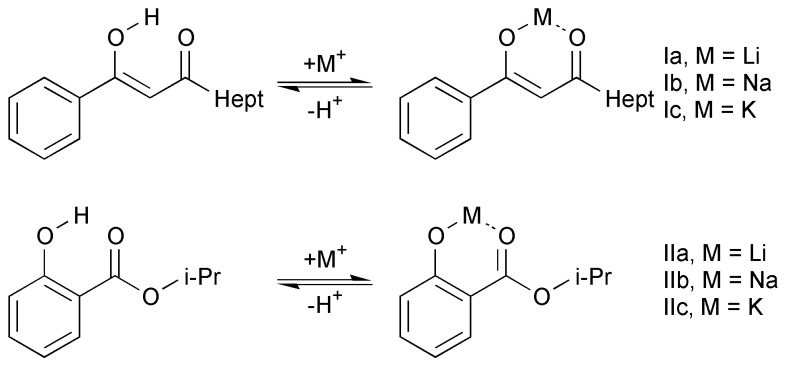
Scheme of formation of the structure of coordination compounds of phenyldecane-1,3-dione (**I**a–c) and isopropyl salicylate (**II**a–c) with lithium, sodium, and potassium cations.

According to Pearson [26], salicylates are hard bases. It could be expected that they form coordination chelates with hard acids such as cations of alkali and alkaline-earth metals; nevertheless, such data are not reported to date. Relying on their structural and functional similarity, we expected that the isopropyl salicylate ion, like the 1,3-enolate ion, could form various stable six-membered metallocycles with alkali metal cations and thus could be considered as new lithium-selective extractants (Scheme 2).

Therefore, in the present work, we focused on the search for a new extraction system for the extraction of lithium cations from a mixture of alkali metal salts. The main goal of the work was to study the selectivity of extraction as well as to find out the structure of the resulting lithium complexes.

## 2. Results and Discussion

### 2.1. Quantum-Chemical Calculations of Alkali Metals Salicylates

**Figure 1:**
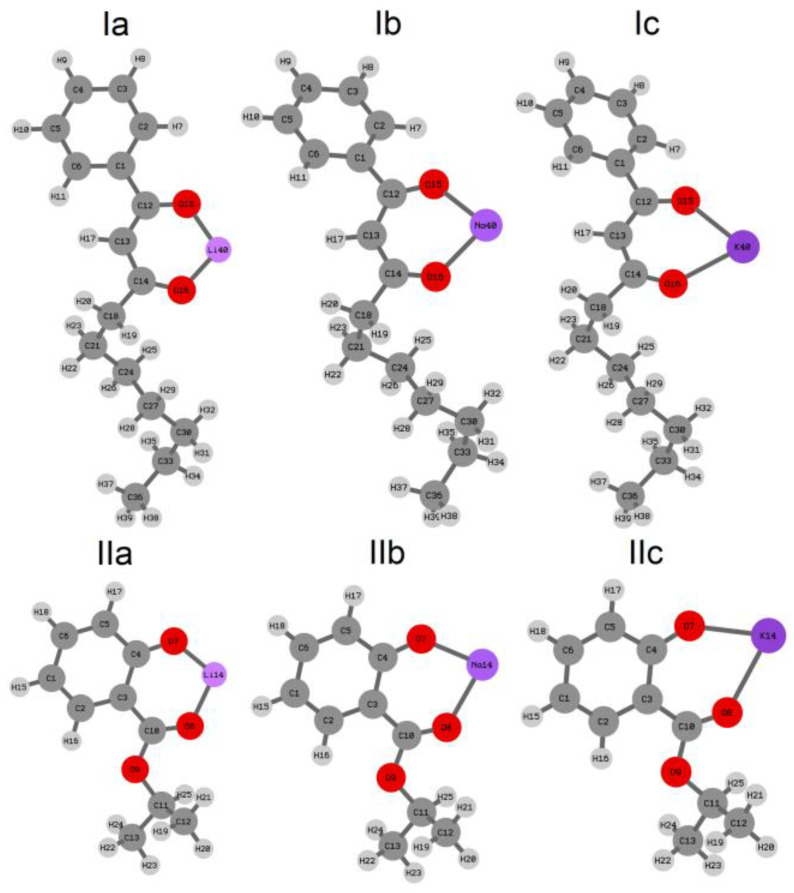
The optimized structure of the complexes of phenyldecane-1,3-dione (**I**) and isopropyl salicylate (**II**) with alkali metals (**a**—Li; **b**—Na; **c**—K) obtained by DFT/B3LYP/6-311 + G (d, p).

In search for fundamental interrelations between the structure of the organic ligand and selective binding of the lithium cation in the presence of sodium and potassium cations, quantum chemical calculations of structural parameters of coordination compounds of phenyldecane-1,3-diketone (I) with cations of lithium (Ia), sodium (Ib), and potassium (Ic) were performed in this work for the first time and shown in Figure 1, Table 1, and in Table S1 in the Supporting Information. The calculation results explain that the preferential binding of the lithium cation by phenyldecane-1,3-diketone (I) as compared with cations of sodium and potassium is attributed to the increasing stability of the obtained metallacycles in the series Ia > Ib > Ic.

The calculation results for the structure of coordination compounds of isopropyl salicylate (II) with cations of lithium (IIa), sodium (IIb), and potassium (IIc) are represented in Figure 1, Table 1, and in Table S2 in the Supporting Information. The bond lengths of the metal-oxygen phenol group and metal-oxygen carboxyl group, as well as in the sequence Ia < Ib < Ic, increase in the series IIa < IIb < IIc. The valence angles in compounds IIa-c are 103° for O_11_-Li_25_-O_12_, 85.62° for O_11_-Na_25_-O_12_ and 72.39° for O_5_-K_11_-O_13_, almost the same as for compounds Ia-c: O_15_-Li_40_-O_16_ = 106.57°, O_15_-Na_40_-O_16_ = 88.82° and O_16_-K_40_-O_15_ = 74.32°. The dihedral angles in metallacycles Ia-c and IIa-c manifest a common tendency: from Li to K the metallacycle plane becomes twisted. These data combined reveal analogous constructions of ligands I and II in their coordination with cations of Li, Na, and K that allow to presume the preferential binding of the lithium cation by isopropyl salicylate as compared to the cations of sodium and potassium.

### 2.2. ATR FTIR and Raman Spectroscopy of Alkali Metal Salicylates

Intramolecular hydrogen bonding is expected for isopropyl salicylate (HL) as well as for the enol form molecules of 1,3-diketones. The formation of an intramolecular hydrogen bond usually results in the closing of a six-membered pseudo-ring structure. The chelated OH…X stretching vibration usually gives rise to a broad absorption band in the 3200–2500 cm^−1^ [27]. The presence of two bands ν(OH) 3200 and 3147 cm^−1^ in this range in the IR spectrum of HL suggests the presence of two spatial modifications of the HL molecule [28]. In the Raman spectra, the weak broad band at 3178 cm^−1^ corresponds to ν(OH). Strong hydrogen bonding decreases the stretching mode ν(OH)) wavenumber and increases out-of-plane deformation mode the γ(OH) wavenumber in IR spectra. The band γ(OH) is recorded in the range of 700–800 cm^−1^ in the spectra of such strongly chelated phenols as ortho-hydroxybenzoyls [29]. The band observed at 728 cm^−1^ in IR spectrum of HL can be assigned to γ(OH). The relative position of the bands ν(OH) and γ(OH) in the IR spectrum of HL corresponds to observed wavenumber for ν(OH) and γ(OH) in spectra of phenol and a number of ortho-substituted hydroxybenzene, where strong intramolecular hydrogen bonds leading to the formation of the chelate ring were found [29].

The position of the characteristic bands ν(Ph) in the IR spectrum HL: 1612, 1585, 1501, 1485 cm^−1^ indicates the presence of conjugation of the benzene ring with substituents [27]. In addition, the noticeable intensity of the band at 1585 cm^−1^ (the band becomes of medium intensity) indicates the presence of conjugation with the external group, which is C=O in our case. According to some researchers, the intensity of this band can be a criterion for the conjugacy of a benzene ring with a carbonyl group or some other unsaturated group. The intensity increase of the first two bands due to the conjugation is accompanied by an intensity decrease of the third band at 1501 cm^−1^ [27].

The band ν(C=O) in the IR spectrum is observed at 1665 cm^−1^, which indicates a low-frequency shift of 45 cm^−1^ relative to the corresponding band in the benzaldehyde spectrum. This is the result of a strong intramolecular hydrogen bond. The strong band at 1671 cm^−1^ corresponds to ν(C=O) stretching mode in the Raman spectrum. The formation of a six-membered pseudo-ring leads to the delocalization of π-electrons throughout the systems of conjugation bonds of the HL molecule. The strong band at 1213 cm^−1^ is assigned to ν(C(=O)-OiPr), the strong band at 1087 cm^−1^ is assigned to ν(C(=O)O-iPr). The skeletal isopropyl deformations are assigned at 1103 and 917 cm^−1^. The bands corresponding to these modes are absent or very weak in the Raman spectrum.

The formation of the six-membered pseudo-ring leads to a greater conjugation of the phenolic group with the benzene ring. Not only does the position of the characteristic bands ν(Ph) changes but also the position of the bands of the phenolic group itself. The stretching ν(PhO) make a significant contribution to the very intense band at 1297 cm^−1^.

The band at 1365 cm^−1^ is combined δ(PhOH) + δ_s_(CH_3_), just like the band at 1250 cm^−1^ is combined δ(PhOH) + ν(Ph), the vibrations δ(PhOH) being its main component. Some shift of bands δ(PhOH) to the high-frequency region in comparison with their locations in the spectra of non-substituted phenols is caused by intramolecular hydrogen bonding of phenolic group with the carbonyl group.

IR and Raman spectroscopy data indicate that the HL molecule is a composition of two conjugated unsaturated six-membered rings; the first one is benzene, and the other is a pseudo-ring formed due to strong intramolecular hydrogen bonds.

**Figure 2:**
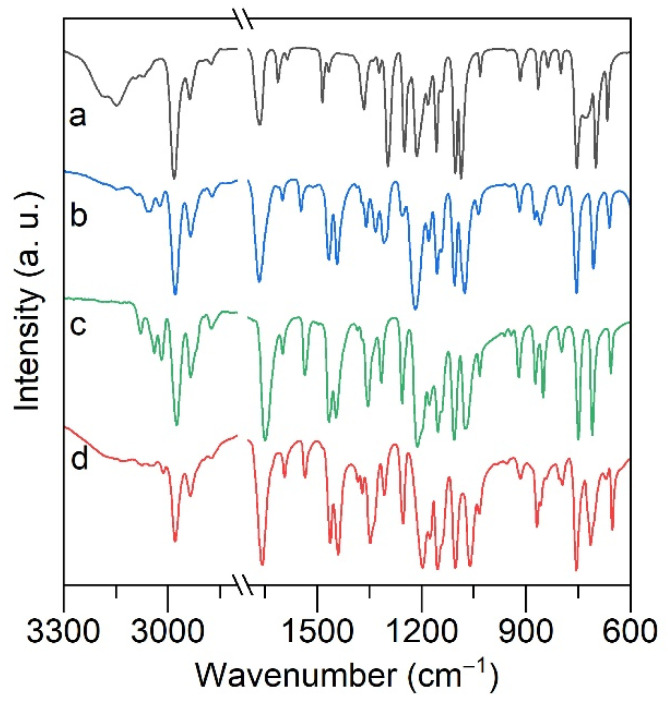
FT-IR spectra: a—isopropyl salicylate (HL), b—LiL, c—NaL, d—KL.

**Figure 3:**
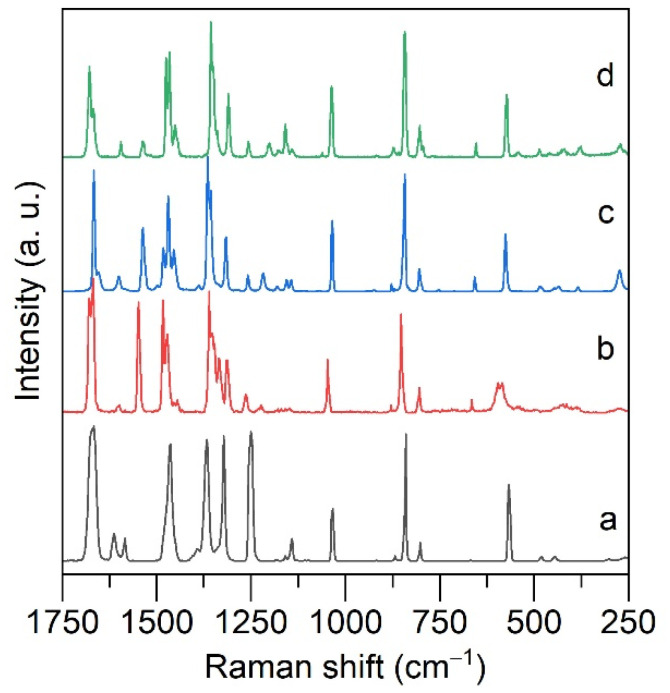
Raman spectra: a—isopropyl salicylate (HL), b—LiL, c—NaL, d—KL.

A comparative analysis of the spectra of HL and alkali isopropyl salicylates (LiL, NaL, KL) allows us to draw conclusions about the structure of the complexes. The six-member pseudo cycle HL is transformed into a real six-member metallocycle. This leads to a significant redistribution of electron density as in the substituents of the benzene ring (OH, C(=O)-O-iPr), and in the benzene ring itself. The formation of a system of conjugated bonds involving two unsaturated rings leads to a change in the IR and Raman spectra (Figure 2 and Figure 3 and full-size spectra Figures S1–S8 in the Supporting Information).

In the IR spectra of the complexes, the absence of bands at 3200 and 3137 cm^−1^, which characterize ν(OH) in the HL molecule suggests deprotonation of the phenol group in the complexes. It also accounts for the disappearance of the band at 728 cm^−1^ γ(OH) and weakening of the relative intensity of the bands at 1250 cm^−1^ (δ(PhOH) + ν(Ph)) and 1365 cm^−1^ (δ(PhOH) + δ_s_(CH_3_)) in the IR spectra of all the complexes. In the Raman spectra of HL, the band at 1250 cm^−1^ has an outstanding intensity, which can be related to δ(PhOH). It is remarkable that the above intense band almost disappears in the Raman spectra of the complexes, and only much less intense bands can be found in this area.

The most significant and characteristic changes in the spectra of the complexes are observed for the very strong band ν(PhO). The formation of a six-membered metallacycle leads to a redistribution of electron density throughout the formed ring conjugated with the benzene ring. This causes strengthening of the bond Ph-O (according to the calculation data) and, consequently, to a shift of the stretching band ν(PhO) 1297 cm^−1^ (HL) to the high-frequency region to 1333, 1309 cm^−1^ (LiL), 1315 cm^−1^ (NaL), 1308 cm^−1^ (KL). At the same time, the relative intensity of the bands weakens in the spectra of all the complexes.

Besides the deprotonated phenol group, the oxygen atom of the carbonyl group participates in the formation of the metallocycle. Since the formation of the coordination bond of the metal with the oxygen of the carbonyl group is accompanied by the destruction of the strong intramolecular hydrogen bond, the position of the band ν(C=O) 1663 cm^−1^ (HL) changes slightly in the spectra of the complexes: 1666 cm^−1^ (LiL), 1649 cm^−1^ (NaL), 1657 cm^−1^ (KL). It is incorrect to draw conclusions about the strength of the formed complexes relying only on the shift of the bands ν(C=O) and ν(PhO). According to quantum chemical calculations, the conformational stability of the resulting metal cycle plays an important role in this case. The shifts of the bands ν(C(=O)-O) and ν(O-iPr) in the spectra of the complexes relative to these bands in the HL spectrum (Figure 2) means the participation of the electrons of the ether oxygen atom in the stabilization of the metallocycle.

Since the complex formation process is accompanied by a redistribution of the electron density over the entire system of conjugated bonds, including two six-membered rings (the benzene ring and the metallocycle), a change in the position of the bands ν(Ph) is recorded in the spectra of the complexes. The position of the characteristic bands ν(Ph) in the spectra of the complexes LiL: 1600, 1548, 1507, 1444 cm^−1^; NaL: 1599, 1536, 1446 cm^−1^, KL: 1593, 1535, 1440 cm^−1^. The shift to the low-frequency region of the band 1585 cm^−1^ (HL) and its increased intensity in the spectra of the complexes indicate an increased conjugation of the benzene ring with the carbonyl group of the substituent.

A greater shift of the ν(Ph) bands to the low-frequency region in the spectra of the complexes, which are even greater than in the spectra of free ligand in relation to the analogous bands in the spectra of non-substituted phenol indicates a greater conjugation of two cyclic moieties: the benzene ring and the metallacycle. The greatest changes in the position of the ν(Ph) bands are observed in the spectrum of KL, the smallest ones in the spectrum of LiL (the smallest deviation from a planar six-membered cycle). The tendency of conjugation getting stronger from a free ligand to complexes and from the lithium complex to the complexes of sodium and potassium is clearly seen in the Raman spectra, where the bands characterizing the benzene ring are clearly defined (Figure 2 and Figure 3).

Analysis of IR and Raman spectra of HL and complexes of alkali metals with it allow to conclude the conjugation of two six-membered cycles: of benzene and a formed metallacycle. The phenolic and carbonyl groups are involved in the formation of the metallocycle, while the ether oxygen atom plays an important role in stabilizing the formed metallocycle by donating electron density. It is shown that in the Li-Na-K series, the stabilization of the metallocycle requires an increase in the degree of electron density redistribution over the entire ligand, including the ether fragment. The obtained data allow us to assume that the complex with lithium is more stable than those with potassium and sodium.

### 2.3. Solvent Extraction

**Figure 4:**
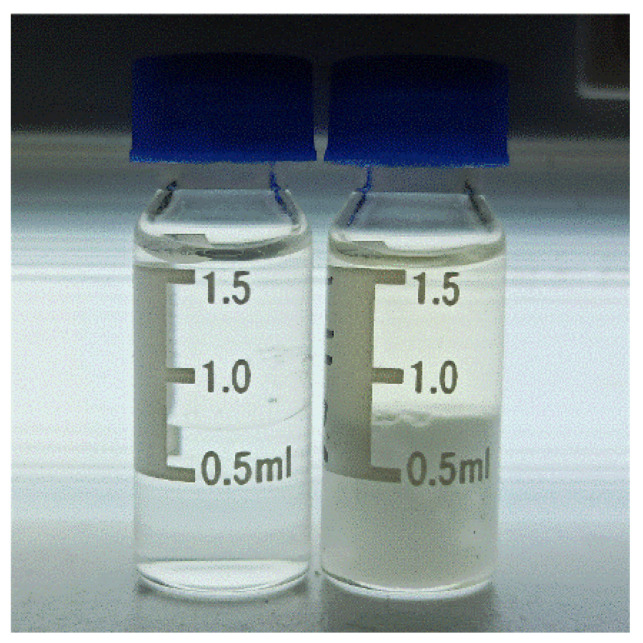
The extraction systems: based on isopropyl salicylate in heptane on the **right** and with addition of TOPO on the **left**.

Extraction of alkali cations from alkaline solutions using HL solution in heptane causes mixture precipitation of hydrated alkali isopropyl salicylates, which are poorly soluble in the organic phase (Figure 4).

**Figure 5:**
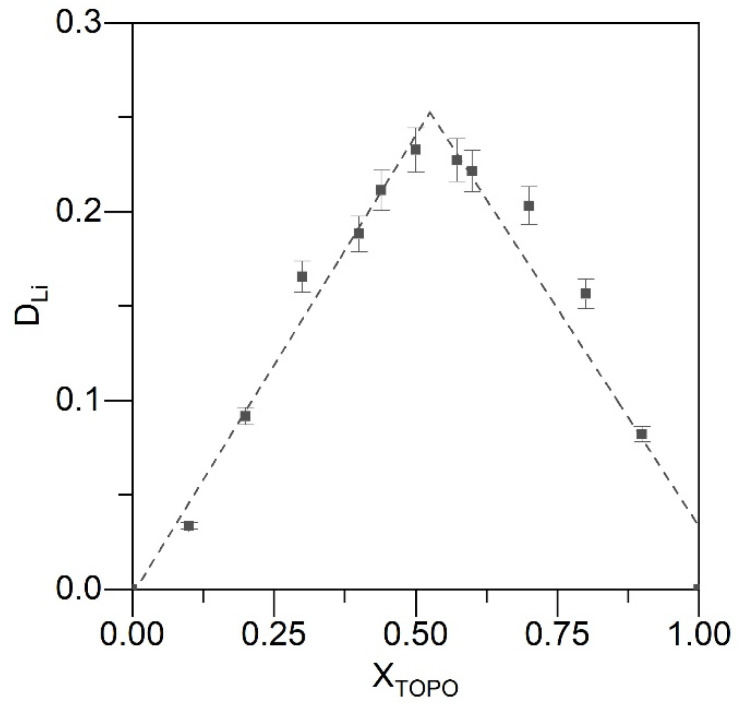
Influence of mole fraction of TOPO on lithium extraction. O/A = 1:1, C_Li_ = 0.02 mol L^−1^, C_NaOH_ = 0.1 M.

Introducing trioctylphosphine oxide (TOPO) as an electron-donor lipophilic additive prevents precipitation. In order to establish the optimal ratio HL-TOPO in the extraction system, we examined the dependence of the lithium distribution coefficient on the mole fraction of TOPO (X_TOPO_) under a constant combined concentration of HL and TOPO (0.15 mol L^−1^). As it can be seen in Figure 5, single components virtually do not extract lithium while their combination gives a strong synergy for lithium extraction. The greatest distribution coefficient for lithium is at X_TOPO_ = 0.5 (the ratio is 1:1). The same behavior is observed for the closest analogue among 1,3-diketones [19].

**Figure 6:**
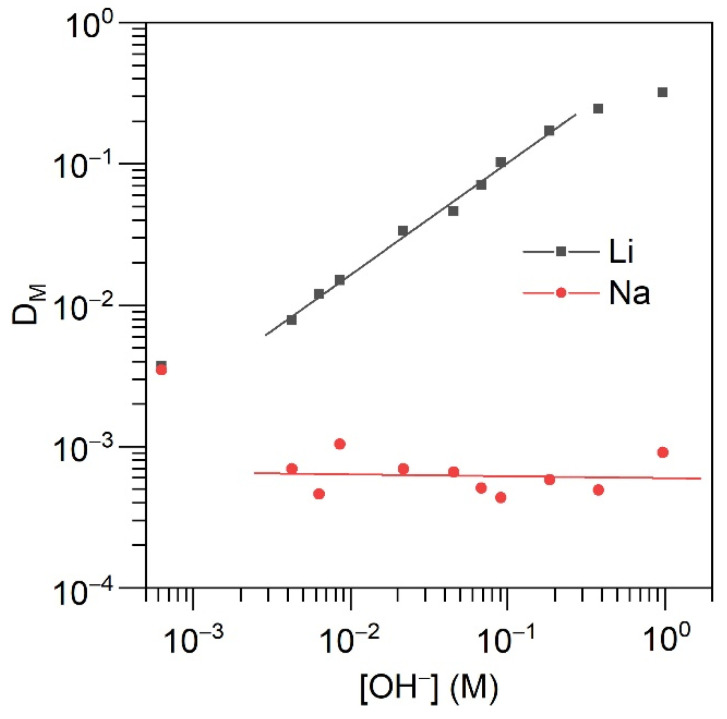
Influence of [OH^−^] on lithium extraction. Aqueous phase: [Li^+^] = 0.5 g L^−1^; organic phase: [HL] = 0.05 mol L^−1^, [TOPO] = 0.05 mol L^−1^; temperature = 25 °C. O/A = 1/1.

The obtained dependence of distribution coefficients of D_Li_, D_Na_ on the equilibrium concentration of hydroxide ions in the above extraction system (Figure 6) shows that the D_Li_ increase as increase the concentration of hydroxide ions, with D_Na_ being unchanged.

As it is shown from the dependence of distribution coefficients on the concentration of hydroxide ions (Figure 6), the extractant works in an alkaline media, so that the re-extraction proceeds readily and complete when a neutral or weakly acidic pH of the rinse water is maintained.

**Figure 7:**
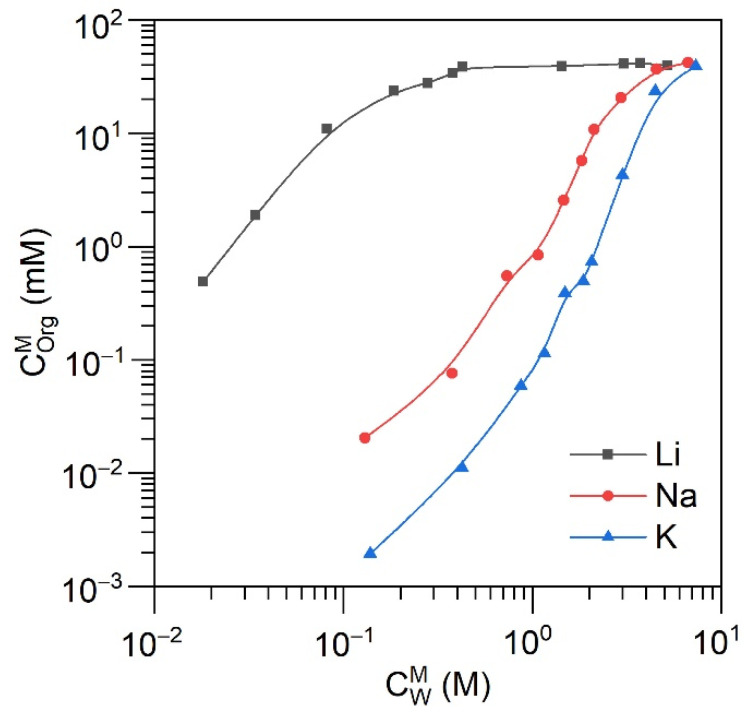
Extraction isotherms. The organic phase consists of 0.05 M salicylate and 0.05 M TOPO in heptane at 25 °C. O/A = 1:1.

The extraction isotherms (Figure 7) with an analogous extraction mixture of water solutions of LiOH, NaOH, and KOH demonstrate that lithium is extracted best of all, and sodium and potassium are extracted to a much lesser degree. Thus, it can be supposed that in the combination of lithium, sodium, and potassium the organic phase will be enriched by lithium and the aqueous phase will be enriched by sodium and potassium.

For a further lithium selectivity study of isopropyl salicylate-based mixture, a model solution containing a small amount of lithium and large amounts of sodium and potassium was prepared (Table 2). The results of extraction experiments (Table 3) show a high lithium selectivity of the isopropyl salicylate-based mixture.

### 2.4. ATR FTIR- and NMR-Spectroscopy of Extracts, Quantum Chemical Calculations of Lithium Salicylate with TOPO

**Figure 8:**
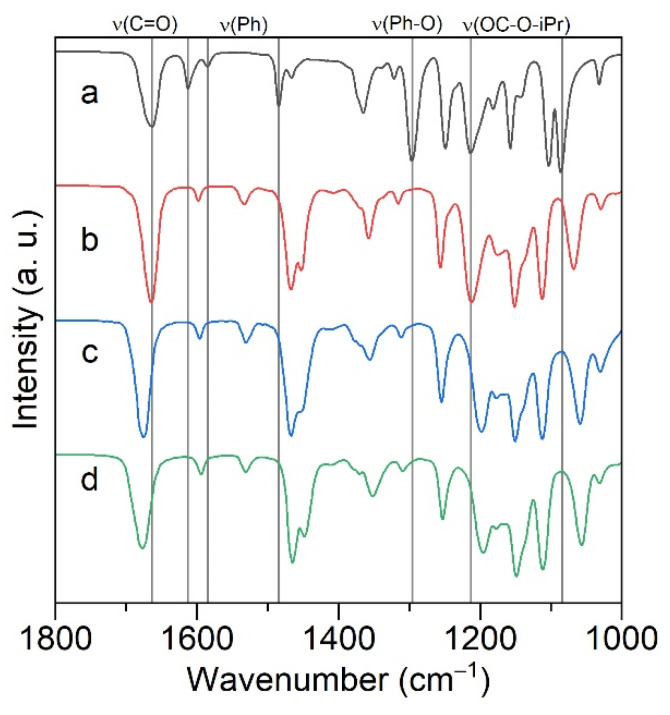
IR-spectra a—HL(L), b—Li(L)TOPO, c—Na(L)TOPO, d—K(L)TOPO.

The IR spectra of the extracts of Li, Na, and K with isopropyl salicylate in the presence of trioctylphosphine oxide (M(L)TOPO) in the most illustrative range of 1000–1800 cm^−1^ are shown in Figure 8 (full-size spectra Figures S9–S12 in the Supporting Information), and the positions of some characteristic bands are given in Table 4.

In the IR spectra of extracts, containing M(L)TOPO complexes, shifts of the ν(Ph) bands to the low-frequency region relative to the same bands in the ML spectra are recorded. This is caused by the redistribution of the electron density in the benzene ring occurring with the entrance of the TOPO molecule into the complex. The presence of a TOPO molecule in the complex leads to bands shifts of the phenol group ν(Ph-O) and the ester group ν(C=O), ν(C(=O)-O), ν(O-iPr). It should be noted that the shift of the bands ν(C(=O)-O) and ν(O-iPr) to the low-frequency region are increases in the Li(L)TOPO–Na(L)TOPO–K(L)TOPO series. This may be because the steric tension of the metallacycles in the case of Na and K is compensated by a greater shift of electron density from the ether moiety, which lengthens the bonds C-O-iPr.

**Figure 9:**
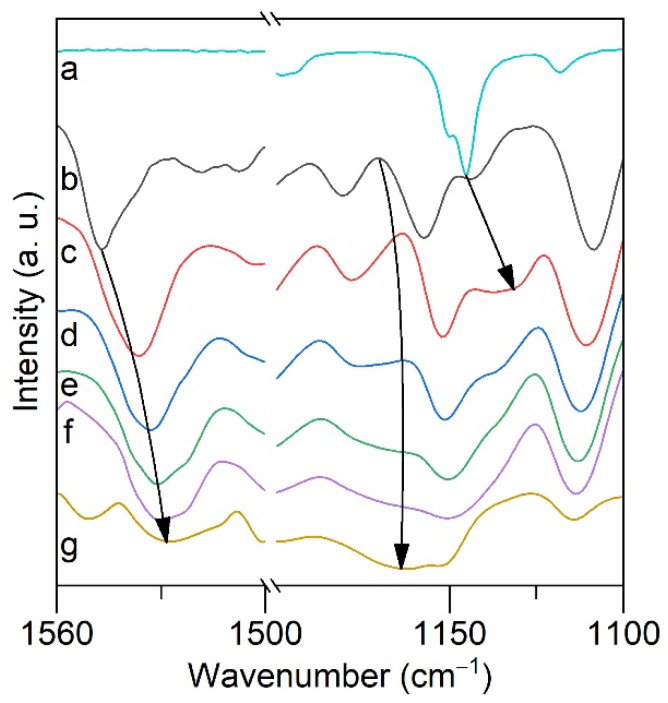
IR-spectra a—trioctylphosphine oxide (TOPO), b—isopropyl salicylate (HL), c—lithium extract of HL:TOPO 2:1, d—HL:TOPO 1:1, e—HL:TOPO 1:2, f—HL:TOPO 1:4, g—HL:TOPO 1:10.

To find out the role of TOPO in the extraction process, IR and NMR spectroscopy were used to investigate organic phases (extracts) with different HL:TOPO ratios. The range of 1200–1125 cm^−1^ was analyzed in the IR spectra (Figure 9). The superimposition of the band ν(P=O) 1145 cm^−1^ on the intense band p(CH_3_) 1157 cm^−1^ makes it hard to judge the coordination of the phosphoryl group. For the HL-TOPO ratio (1:0.5:1), the band p(CH_3_) exhibits a shoulder at 1137 cm^−1^ as a result of the shift to the low-frequency region of the band ν(P=O).

This may be caused by the formation of a coordination bond Li…O=P. For the concentrations of TOPO more than 1:1, this band widens and a shoulder appears in the high-frequency region at 1168 cm^−1^. This may be explained by the solvation mechanism of binding of TOPO molecules in the lithium complex coming into play. Changes in the spectra in the range 1560–1510 cm^−1^, where ν(Ph) bands are observed, confirm the participation of TOPO molecules in the formation of the overall conjugated system. The shift of the ν(Ph) band to the low-frequency region by 14 cm^−1^ down to 1534 cm^−1^ in the spectrum of Li(L)TOPO as compared to the spectrum of LiL (Table 4) may be explained by the entrance of a TOPO molecule into the composition of the extracted complex and the resultant rebuild of all the entire conjugated system. Further minor but persistent shifts of this band ν(Ph) to the low-frequency region (Figure 9) are due to the formation of solvates. It is evident that the ratio HL:TOPO 1:1 is the boundary between the process of coordination binding of TOPO molecules and the process of solvation by TOPO molecules.

**Figure 10:**
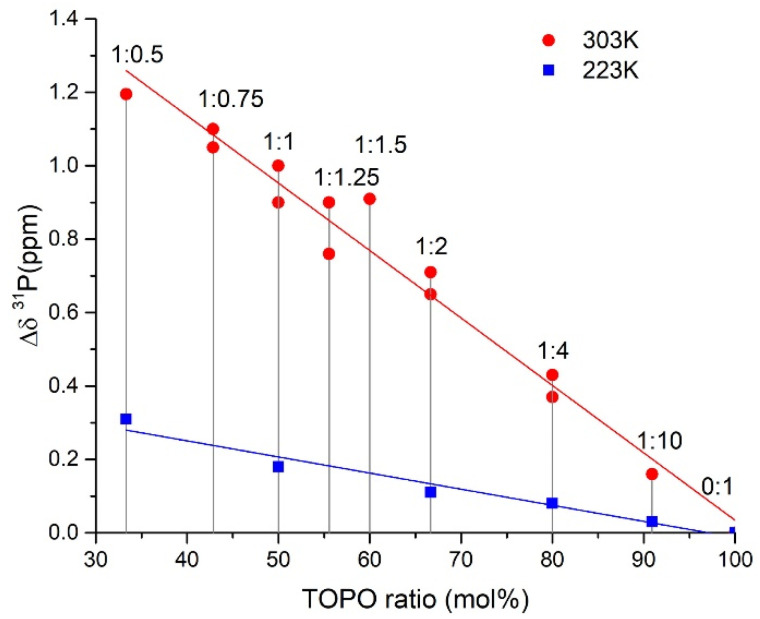
The changes of chemical shifts in ^31^P NMR spectra of Li(L)TOPO upon variation of LiL/TOPO ratio (CDCl_3_).

A series of samples containing LiL and a varied amount of TOPO was investigated by NMR in CDCl_3_ at 303 K and 223 K. In the case of the expected coordination of P=O fragment to lithium ion ^31^P NMR data may provide information on the stoichiometry of the formed adduct. The chemical shift of the phosphorus atom of TOPO in the absence of LiL was used as a reference and the corresponding values in the spectra of adducts were analyzed with respect to it (Figure 10 and Figure S13).

The variation of the LiL ratio at 303 K expectedly results in the shift of the observed ^31^P resonance. Stepwise increase of the molar ratio of LiL results in a gradual downfield shift of the ^31^P signal of TOPO, which could be explained by the interaction with lithium cation and thus by the decrease of the electronic density around the phosphorus atom. The observed chemical shifts fall within a fine linear correlation with the molar ratio of TOPO in the mixture. It should also be noted that in the whole observed range of LiL/TOPO ratio only a single resonance of ^31^P was observed, revealing fast exchange between the associated and dissociated state of TOPO.

In this respect, we attempted to stabilize the Li(L)TOPO adduct at low temperature. Nevertheless, at 223 K single ^31^P resonance was observed in all cases with gradual broadening upon the increase of the ratio of Li-containing component. In this case, the chemical shift of the observed signal is also shifted downfield monotonically while the magnitude of the shift is three times lower.

^1^H NMR data for the investigated series of complexes is considerably less informative in the comparison with ^31^P (Figure S14). At all Li(L)TOPO ratios, the difference in the positions of the observed chemical shifts is negligible and the resonances are broadened. Obviously, the observed resonances are completely different from the ones of free L as a result of the coordination shifts. Nevertheless, it should be mentioned that at 1:1 ratio the resolution of the resonances is enhanced that is in accordance with the evaluated stoichiometry of the adduct. The decrease of the temperature to 223 K results in a significant broadening of all signals that make the spectra noninterpretive.

**Figure 11:**
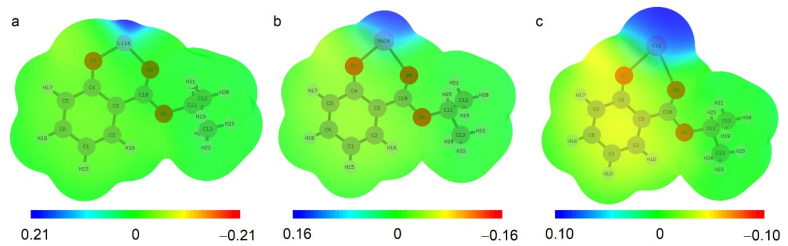
Electron density from the total SCF density (isoval = 0.0004) mapped with the electrostatic potential (ESP) for LiL (**a**), NaL (**b**), and KL (**c**).

Quantum-chemical calculations show an excess of positive charge on lithium in LiL complex (Figure 11). This provides for the possibility of binding electron-donor ligand, namely TOPO in our case. According to NMR and IR spectroscopy and extraction experiments, the greatest possibility of efficient binding of Li^+^ with L^−^ and TOPO exists with the ratio 1:1:1. After finding this ratio, this structure was modeled (Figure S13 in the Supporting Information).

A comparison of geometric parameters of the calculated LiL and Li(L)TOPO complexes shows (Table 5 and Table S3 in Supporting Information) that the coordination of Li to TOPO lead to bonds extension between lithium and phenol oxygen atom (Li-O_1_) and carbonyl oxygen atom (Li-O_2_), while the bonds C=O, Ph-O are slightly affected, which is confirmed by IR spectroscopy of extracts. Results of calculated data for Li(L)TOPO complex and IR spectroscopy of the extract confirm the composition of the extracted complex Li:L:TOPO = 1:1:1.

The localization of excessive negative charge at the benzene ring in the Li(L)TOPO complex (Figure S13 in the Supporting Information) indicates the possibility of p,π-interaction between the benzene ring and the phosphoryl group oxygen atom of TOPO, which results in the formation of solvate shell with the involving TOPO. This leads to a shift of the ν(P=O) band to the high-frequency range in the IR spectra of extracts with an HL-TOPO ratio of more than 1:1.

## 3. Materials and Methods

### 3.1. Quantum Chemical Calculations

The geometric optimization of the compound considered was performed using the DFT functional method (B3LYP) with the basis set 6-311G + (d, p). All the calculations were performed using Gaussian 06 program.

### 3.2. Extraction

The metal salts as well as metal hydroxides and n-heptane were all purchased from Sigma Aldrich and are of the highest purity available. The metal salts are weighed with OHAUS Pioneer (PA214C) (precision ± 0.1 mg) and dissolved in deionized water to prepare the aqueous phase. The acidity of the metal salt solution is adjusted by adding sodium hydroxide. The organic phase is made of isopropyl salicylate and TOPO dissolved in heptane. These two immiscible phases are then shaken for 30 min at 30 rpm at 25 °C with a Biosan Mini-rotator Bio RS-24 apparatus to promote phase mixing. The extraction kinetics under these conditions is very fast, and 1 h of mixing is obviously excessive. The organic to aqueous volume ratio (O/A) is maintained as 1:1. The two phases are then centrifuged at 6000 rpm for 5 min with a Hettich EBA-200 apparatus to promote phase disengagement. The concentration of metal ions in the aqueous phase and organic phase is determined by Agilent 7500ce IPC-MS apparatus.

The distribution ratio for the metal (D_M_) is calculated with the following equation:

$$D_{M}=\frac{\left[M\right]\;\mathrm{in}\;\mathrm{org}}{\left[M\right]\;\mathrm{in}\;\mathrm{water}}$$

[M]_org_ and [M]_aq_ being the metal concentration in the organic and aqueous phase after extraction, respectively.

The separation coefficients between Li^+^ and M^+^ (Na^+^ or K^+^) were calculated with the following equation:

$$\beta_{\mathrm{Li}/M}=\frac{D_{\mathrm{Li}}}{D_{M}}$$

### 3.3. ATR FT-IR Spectra

FT–IR spectra were measured using a JASCO FT/IR-6600 spectrometer on an ATR PRO ONE Technologies attachment with a diamond crystal PKS-D1F by the ATR (attenuated total reflectance) method in the range 4000–250 cm^−1^. Solid samples were placed on the diamond crystal without sample preparation. When analyzing the organic phases (extracts), the preconcentration was performed by removing the solvent under vacuum.

### 3.4. Raman Spectra

Raman spectra were obtained with a Renishaw inVia Reflex Microscope system equipped with a Peltier-cooled CCD. The 633 nm lines of a He–Ne laser and the 405 nm lines of a diode laser were used for excitation. Laser light was focused on the sample through a 50× objective to a spot size of ~2 μm. The power on the sample was <0.1 mW. Spectral resolution is about 2 cm^−1^.

### 3.5. NMR Spectra

The routine NMR spectrum of HL was recorded in CDCl3 on a Bruker CXP-200 spectrometer using TMS as an internal reference.

VT-NMR investigations were recorded using a Bruker Avance III spectrometer with 600.13 MHz proton frequency in CDCl3 at 223 K and 303 K with ±0.1 K accuracy. ^1^H NMR chemical shifts were referenced to the residual solvent resonance as an internal reference, while for ^31^P NMR spectra external standard H_3_PO_4_ was used. The complete NMR spectra are represented in supplementary data.

### 3.6. Synthesis

*Isopropyl salicylate (HL).* A total of 3 mL of sulfuric acid was added to a solution of salicylic acid (13.81 g, 0.01 mmol) in 150 mL of isopropyl alcohol. The mixture was in a reflux condenser for 8 h and isopropyl alcohol was evaporated in vacuo. A total of 150 mL of distilled water was added to the residue and afterward the concentrated solution of sodium hydrocarbonate was added to pH = 7. The obtained mixture was extracted with CHCl_3_ (2 × 50 mL). The combined extracts were washed with distilled water (3 × 50 mL), dried over sodium sulfate, evaporated, and the residue was distilled under reduced pressure. The yield of the compound after redistilling was 12.0 g, T_boiling_ 115–118 °C/10 mm Hg (67%). Found, %: C 65.55; H 6.57. C_10_H_12_O_3_. Calculated, %: C 65.65; H 6.71. 1H NMR spectrum (200 MHz, CDCl_3_, δ, ppm): 1.37 d (6H, ^3^J_H-H_ = 7.46 Hz, 2CH_3_CHO), 5.29 _K_ (2H, ^3^J_H-H_ = 7.78 Hz, CH_3_CHO), 6.88 _T_ (1H, ^3^J_H-H_ = 7.0 Hz, Ar-H), 6.95 d (1H, ^3^J_H-H_ = 7.7 Hz, Ar-H), 7.43 _T_ (1H, ^3^J_H-H_ = 8.5 Hz, Ar-H), 7.83 d (1H, ^3^J_H-H_ = 6.4 Hz, Ar-H), 10.96 c. (1H, Ar-OH).

(c) Isopropyl Salicylate: Yield: 93%; 1H NMR (300 MHz, CDCl_3_): δH 1.34–1.36 (d, J = 6.0 Hz, 6H), 5.19–5.26 (m, 1H), 6.61–6.66 (m, 2H), 7.22–7.28 (m, 1H), 7.85–7.88 (m, 1H), 10.50 (s, 1H); 13C NMR (75 MHz, CDCl_3_) δC 22.0, 67.6, 60 111.6, 116.2, 116.6, 131.3, 133.49, 150.4, 167.7.

Isopropyl salicylate is an oily liquid with a specific odor and is extremely soluble in hydrocarbons. According to SDS, it is an irritant (categories 2, 3) and requires eye and skin protection when working in its pure form. From the point of view of fire safety, isopropyl salicylate is a flammable liquid and must be handled away from open flames.

*Lithium salicylate (LiL).* 2.28 g (8.00 mmol) of lithium bis((trifluoromethyl)sulfonyl)amide was added to 1.55 g (8.60 mmol) of isopropyl salicylate solution in 25 mL of dry dioxane. The mixture was heated to the boiling point and stirred for 15 min and the solvent was evaporated in a vacuum. The hexane (15 mL) was added to the residue and the precipitate was filtered off, which was successively washed with hexane (2 × 25 mL), cold distilled water (5 mL), and dried for 20 h in a vacuum desiccator. The yield of compound IIa was 1.20 g, 81%. Found, %: C 64.39; H 5.85. For C_10_H_11_LiO_3_ anal. calcd., %: C 64.53; H 5.96.

*Sodium salicylate (NaL).* Prepared similarly to compound IIa from 0.35 g, (8.00 mmol) 55% suspension of sodium hydride in paraffin and 1.55 g (8.60 mmol) of the solution of isopropyl salicylate in 55 mL of dry dioxane. The yield of compound IIb was 1.13 g, 70%. Found, %: C 57.91; H 5.70. For C_10_H_11_NaO_3_ anal. calcd., %: C 59.41; H 5.48.

*Potassium salicylate (KL).* Prepared similarly to compound IIa from 0.90 g (8.00 mmol) of potassium tert-butylate and 1.55 g (8.60 mmol) of the solution of isopropyl salicylate in 40 mL of dry dioxane. The yield of compound IIc was 1.17 g, 67%. Found, %: C 54.90; H 4.93. For C_10_H_11_KO_3_ anal. calcd., %: C 55.02; H 5.08.

## 4. Conclusions

DFT-calculations showed that both isopropyl salicylate (HL) and 1,3-diketone form six-membered metallacycles with cations of alkali metals. The analysis of the geometry of the metallacycles indicates the preferential binding of lithium cation by isopropyl salicylate as compared with sodium and potassium cations.

An analysis of IR and Raman spectra of complexes of alkali cations with HL demonstrates the conjugation of two six-membered cycles, namely the benzene ring and the formed metallacycle. The oxygen of the phenolic and carbonyl groups participates in the formation of the metallacycle while the ester oxygen atom plays an important role in the stabilization of the formed metallacycle by providing electron density. It was shown that in the series Li-Na-K, stabilization of the metallacycle requires greater redistribution of electron density throughout the ligand, including the ether moiety. The obtained data clearly indicate the lithium complex to be more stable than those of potassium or sodium.

The extraction ability of isopropyl salicylate is manifested in the presence of electron-donor additive trioctylphosphine oxide (TOPO). The greatest distribution coefficient for lithium is achieved at X_TOPO_ = 0.5 (ratio 1:1). Similar behavior is observed for the closest analogue among 1,3-diketones [19]. The extraction occurs under highly alkaline conditions. The results of the extraction experiments show a high lithium selectivity of the mixture based on isopropyl salicylate.

According to DFT calculations and NMR and IR spectroscopy, the most probable composition of the extracted lithium complex has the ratio Li:L:TOPO = 1:1:1. The additional introduction of TOPO leads to solvation.

## Figures and Tables

**Table 1:** Bond lengths (Å) and angles (°) in six-membered metallacycles Ia-c and IIa-c.

	M-O_1_	M-O_2_ *	O_1_-M-O_2_
Ia	1.775	1.781	106.55
Ib	2.134	2.140	88.83
Ic	2.446	2.454	74.26
IIa	1.733	1.805	103.99
IIb	2.088	2.163	84.86
IIc	2.374	2.505	71.06

*—carbonyl oxygen atom for II.

**Table 2:** Composition of the model solution (concentration ions g/L).

Li^+^	Na^+^	K^+^
0.455	37.9	107

**Table 3:** Lithium distribution coefficient, separation coefficients Li/Na and Li/K.

C_HL+TOPO_ mol L^−1^	D_Li_	D_Na_	D_K_	β_Li/Na_	β_Li/K_
0.2	6.2500	0.0042	0.0008	1.5·10^3^	7.6·10^3^

**Table 4:** Some bands in the IR spectra for HL and complexes of alkali metals (cm^−1^).

	ν(C=O)	ν(Ph)	ν(Ph-O)	ν(OC-OiPr)	ν(OCO-iPr)
HL	1665	1613, 1585, 1485	1300	1213	1085
LiL	1668	1600, 1548, 1444	1309, 1332	1222	1078
Li(L)TOPO	1664	1598, 1533, 1453	1316	1212	1067
NaL	1651	1599, 1536, 1446	1315	1212	1075
Na(L)TOPO	1676	1596, 1531, 1452	1311	1199	1059
KL	1658	1600, 1547, 1443	1308	1200	1064
K(L)TOPO	1677	1595, 1531, 1448	1309	1196	1057

**Table 5:** Binding lengths values di (Å) and angles (°) in six-membered metallacycles LiL and Li(L)TOPO.

	Li-O_1_	Li-O_2_	Ph-O_1_	C=O_2_
LiL	1.745	1.804	1.289	1.248
Li(L)TOPO	1.817	1.864	1.285	1.238

## Data Availability

Not applicable.

## Supplementary Materials

The following supporting information can be downloaded at: https://www.mdpi.com/article/10.3390/molecules27103051/s1, Figure S1: FT-IR spectrum of HL (II); Figure S2. FT-IR spectrum of LiL (IIa); Figure S3. FT-IR spectrum of NaL (IIb); Figure S4. FT-IR spectrum of KL (IIc); Figure S5. Raman spectrum of HL (II); Figure S6. Raman spectrum of LiL (IIa); Figure S7. Raman spectrum of NaL (IIb); Figure S8. Raman spectrum of KL (IIc); Figure S9. FT-IR spectrum of TOPO; Figure S10. FT-IR spectrum of Li(L)TOPO (IIa); Figure S11. FT-IR spectrum of Na(L)TOPO (IIb); Figure S12. FT-IR spectrum of K(L)TOPO (IIc); Figure S13. 31P NMR spectra of the Li(L)TOPO system at various component ratio (303K, CDCl3); Figure S14. 1H NMR spectra of the Li(L)TOPO system at various component ratios (CDCl3); Figure S15. Electron density from the total SCF density (isoval = 0.0004) mapped with the electrostatic potential (ESP) for Li(L)TOPO; Table S1: Geometric characteristics of complexes alkali metal with phenyldecane-1,3-dione (Ia—Li, Ib—Na, Ic—K) obtained by DFT calculations (B3LYP 6-311 + G(d,p)). Table S2. Geometric characteristics of alkali metal isopropyl salicylates (IIa—Li, IIb—Na, IIc—K) obtained by DFT calculations (B3LYP 6-311 + G(d,p)); Table S3. Geometric characteristics of alkali metal isopropyl salicylates (IIa) and adduct isopropyl salicylates with TOPO (Li(L)TOPO) obtained by DFT calculations (B3LYP 6-311 + G(d,p)); Table S4. Variation of 13P chemical shifts at different IPSAL-Li/TOPO ratio.

## Sample Availability

Samples of the compounds are available from the authors.
