# Supplementary material for: A New Extraction System Based on Isopropyl Salicylate and Trioctylphosphine Oxide for Separating Alkali Metals
Source: Molecules. 2022 May 10;27(10):3051. doi: 10.3390/molecules27103051 (PMC9146891; doi:10.3390/molecules27103051)
Supplement: Supplementary file 1 [file molecules-27-03051-s001.zip › molecules-1696153-supplementary.pdf]

Appendix A. Supplementary data
A new extraction system based on isopropyl salicylate and trioctylphosphine oxide for separating alkali metals

A.Y. Tsivadze, A.A. Bezdomnikov, V.E. Baulin, L.I. Demina, D.V. Baulin, Y.I. Rogacheva, K.P. Birin*

Laboratory of Novel Physicochemical Problems, A.N. Frumkin Institute of Physical Chemistry and Electrochemistry RAS, Moscow, Russian Federation, 119071

**Corresponding author:*
Alexey A. Bezdomnikov
email: bezdomnikovaa@phyche.ac.ru

Contents:

Table S1. Geometric characteristics of complexes alkali metal with phenyldecane-1,3-dione (Ia – Li, Ib – Na, Ic – K) obtained by DFT calculations (B3LYP 6-311+ G(d,p)).....3

Table S2. Geometric characteristics of alkali metal isopropyl salicylates (IIa – Li, IIb – Na, IIc – K) obtained by DFT calculations (B3LYP 6-311+ G(d,p)). 4

Table S3. Geometric characteristics of alkali metal isopropyl salicylates (IIa) and adduct isopropyl salicylates with TOPO (Li(L)TOPO) obtained by DFT calculations (B3LYP 6-311+ G(d,p))...5

Figure S1. FT-IR spectrum of HL (II). 6

Figure S2. FT-IR spectrum of LiL (IIa). 6

Figure S3. FT-IR spectrum of NaL (IIb). 7

Figure S4. FT-IR spectrum of KL (IIc)..7

Figure S5. Raman spectrum of HL (II)...8

Figure S6. Raman spectrum of LiL (IIa)...8

Figure S7. Raman spectrum of NaL (IIb)...9

Figure S8. Raman spectrum of KL (IIc). 9

Figure S9. FT-IR spectrum of TOPO. 10

Figure S10. FT-IR spectrum of Li(L)TOPO (IIa)..10

Figure S11. FT-IR spectrum of Na(L)TOPO (IIb)...11

Figure S12. FT-IR spectrum of K(L)TOPO (IIc). 11

Table S4. Variation of 13P chemical shifts at different IPSAL-Li / TOPO ratio.............12

Figure S13. ^{31}P NMR spectra of the Li(L)TOPO system at various component ratio (303K, $CDCl_3$)...12

Figure S14. 1H NMR spectra of the Li(L)TOPO system at various component ratios ($CDCl_3$). 13

Figure S15. Electron density from the total SCF density (isoval = 0.0004) mapped with the electrostatic potential (ESP) for Li(L)TOPO...13

Table S1. Geometric characteristics of complexes alkali metal with phenyldecane-1,3-dione (Ia – Li, Ib – Na, Ic – K) obtained by DFT calculations (B3LYP 6-311+ G(d,p)).

	Ia	Ib	Ic
	Bond length. Å		
C1-C12	1.503	1.510	1.512
C12-C13	1.412	1.414	1.414
C13-C14	1.413	1.417	1.417
C14-C18	1.520	1.527	1.529
C12-O15	1.274	1.268	1.265
C14-O16	1.272	1.266	1.262
O15-M40	1.775	2.134	2.446
O16-M40	1.781	2.140	2.454
C13-H17	1.081	1.081	1.082
	Valence angle. °		
C12-C13-C14	125.726	127.917	127.186
O15-C12-C13	124.244	125.827	125.716
M40-O15-C12	119.546	125.621	133.302
C13-C14-O16	125.159	126.531	126.245
M40-O16-C14	118.677	125.015	132.869
O16-M40-O15	106.553	88.829	74.259
	Dihedral angle. °		
O16-C14-O15-Li40	-1.117	-0.907	-0.993
O16-C14-C12-O15	-2.659	3.558	4.378

Table S2. Geometric characteristics of alkali metal isopropyl salicylates (IIa – Li, IIb – Na, IIc – K) obtained by DFT calculations (B3LYP 6-311+ G(d,p)).

	IIa	IIb	IIc
	Bond length. Å		
C1-C2	1.376	1.375	1.377
C2-C3	1.419	1.420	1.419
C3-C4	1.448	1.453	1.453
C4-C5	1.430	1.436	1.437
C5-C6	1.374	1.373	1.373
C6-C1	1.411	1.411	1.411
C3-C10	1.453	1.457	1.457
C4-O7	1.289	1.281	1.279
C10-O8	1.248	1.241	1.237
C10-O9	1.345	1.357	1.362
O9-C11	1.459	1.452	1.450
O7-M14	1.733	2.088	2.374
O8-M14	1.805	2.163	2.505
C2-H16	1.082	1.082	1.082
C5-H17	1.084	1.084	1.085
H16-O9	2.311	2.269	2.276
H17-O7	2.548	2.536	2.547
	Valence angle. °		
C4-C3-C10	121.157	122.058	121.526
O7-C4-C3	124.370	125.207	124.744
M14-O7-C4	123.086	131.555	140.166
C3-C10-O8	126.296	127.463	127.397
M14-O8-C10	121.099	128.846	135.089
O7-M14-O8	103.990	84.863	71.062
	Dihedral angle. °		
C1-C3-C4-M14	-179.928	-179.629	-179.124
C6-C4-C3-O8	-179.869	-179.938	179.918
O8-C10-O7-M14	0.248	0.516	0.618
O8-C10-C4-O7	0.136	0.118	0.318
C2-C1-C6-C5	0.022	0.036	0.021

Table S3. Geometric characteristics of alkali metal isopropyl salicylates (IIa) and adduct isopropyl salicylates with TOPO (Li(L)TOPO) obtained by DFT calculations (B3LYP 6-311+ G(d,p)).

	LiL (IIa)	Li(L)TOPO
	Bond length. Å	
C1-C2	1.376	1.378
C2-C3	1.419	1.416
C3-C4	1.448	1.448
C4-C5	1.430	1.433
C5-C6	1.374	1.375
C6-C1	1.411	1.410
C3-C10	1.453	1.457
C4-O7	1.289	1.285
C10-O8	1.248	1.238
C10-O9	1.345	1.352
O9-C11	1.459	1.456
O7-M14	1.733	1.817
O8-M14	1.805	1.864
C2-H16	1.082	1.082
C5-H17	1.084	1.085
H16-O9	2.311	2.309
H17-O7	2.548	2.557
	Valence angle. °	
C4-C3-C10	121.157	120.676
O7-C4-C3	124.370	124.462
M14-O7-C4	123.086	125.873
C3-C10-O8	126.296	126.424
M14-O8-C10	121.099	124.586
O7-M14-O8	103.990	97.958
	Dihedral angle. °	
C1-C3-C4-M14	-179.928	-179.389
C6-C4-C3-O8	-179.869	-179.852
O8-C10-O7-M14	0.248	0.694
O8-C10-C4-O7	0.136	0.392
C2-C1-C6-C5	0.022	0.009

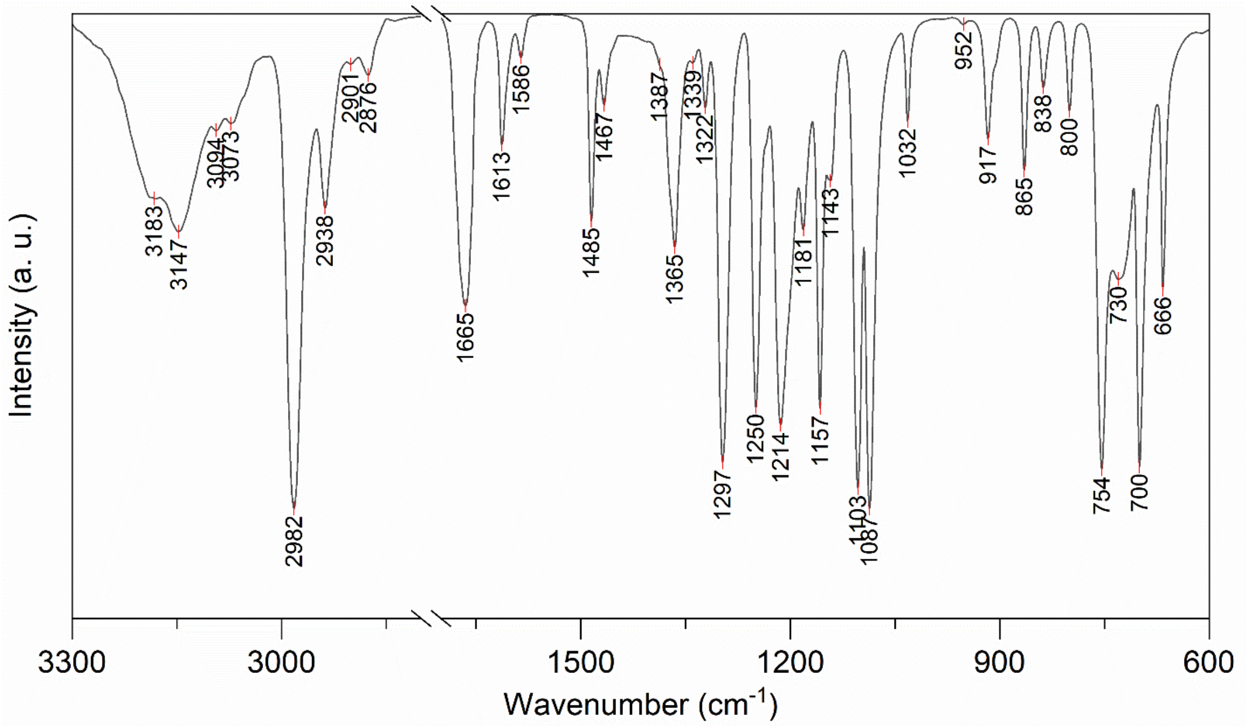

Figure S1. FT-IR spectrum of HL (II).

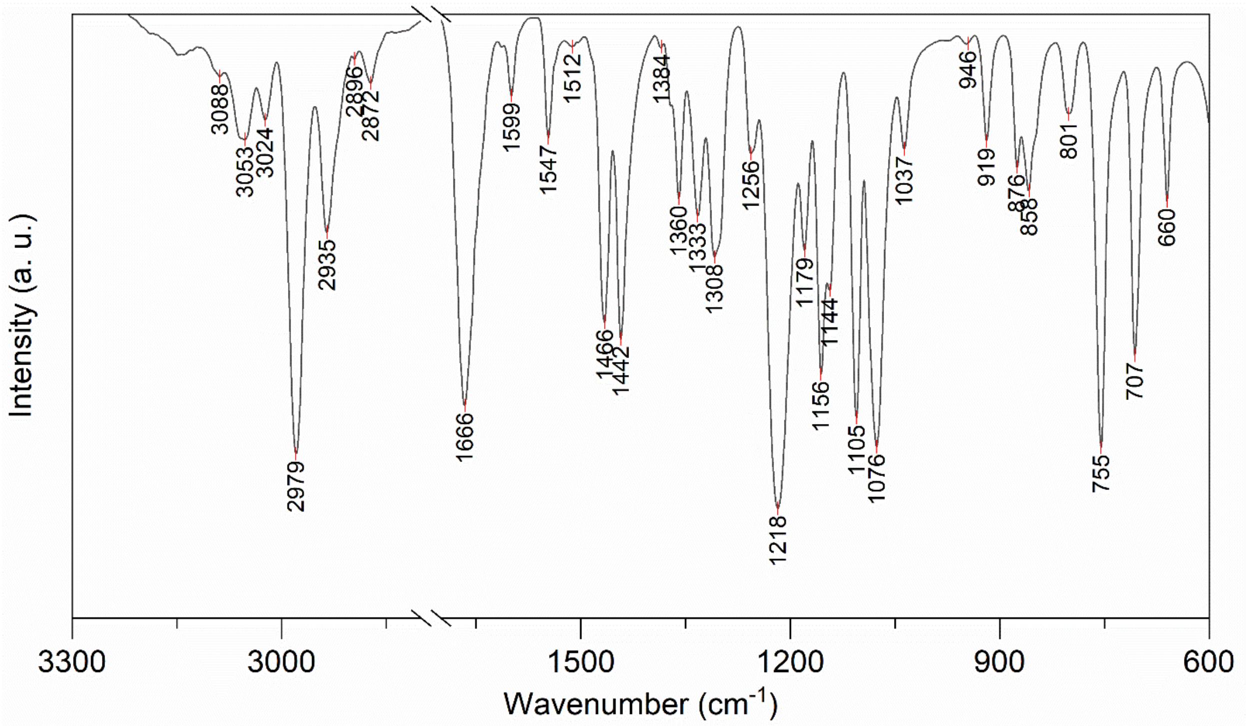

Figure S2. FT-IR spectrum of LiL (IIa).

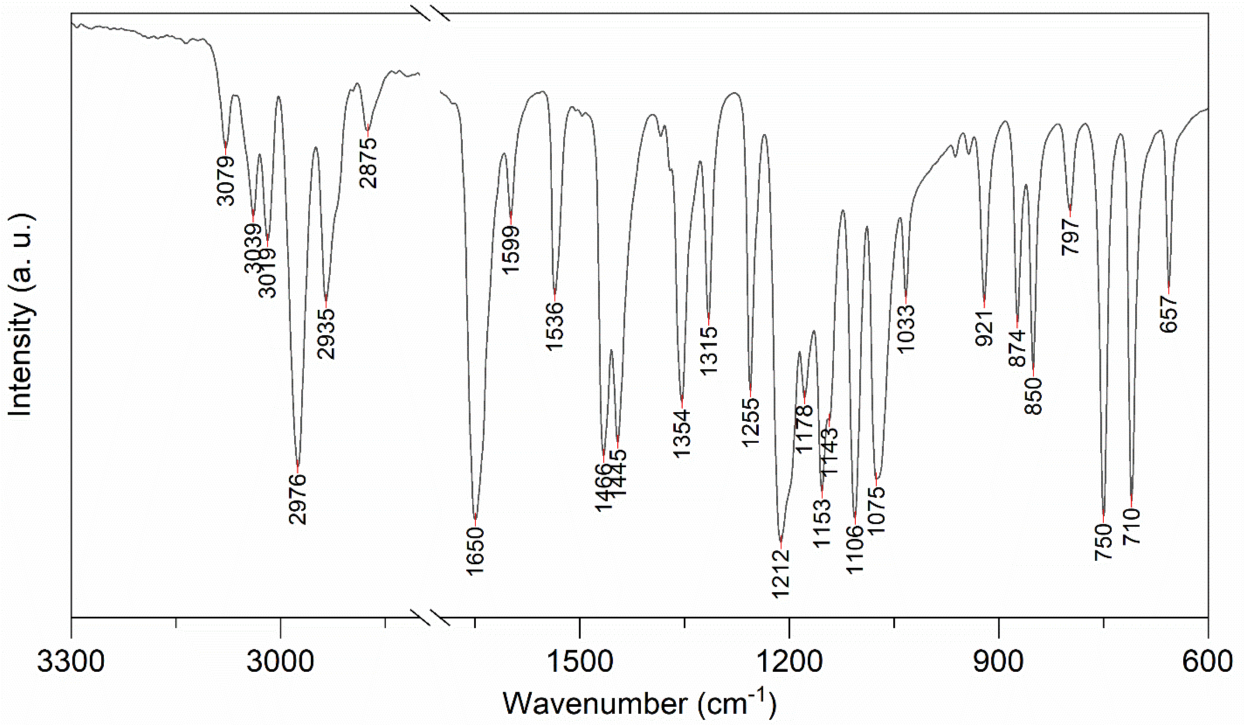

Figure S3. FT-IR spectrum of NaL (IIb).

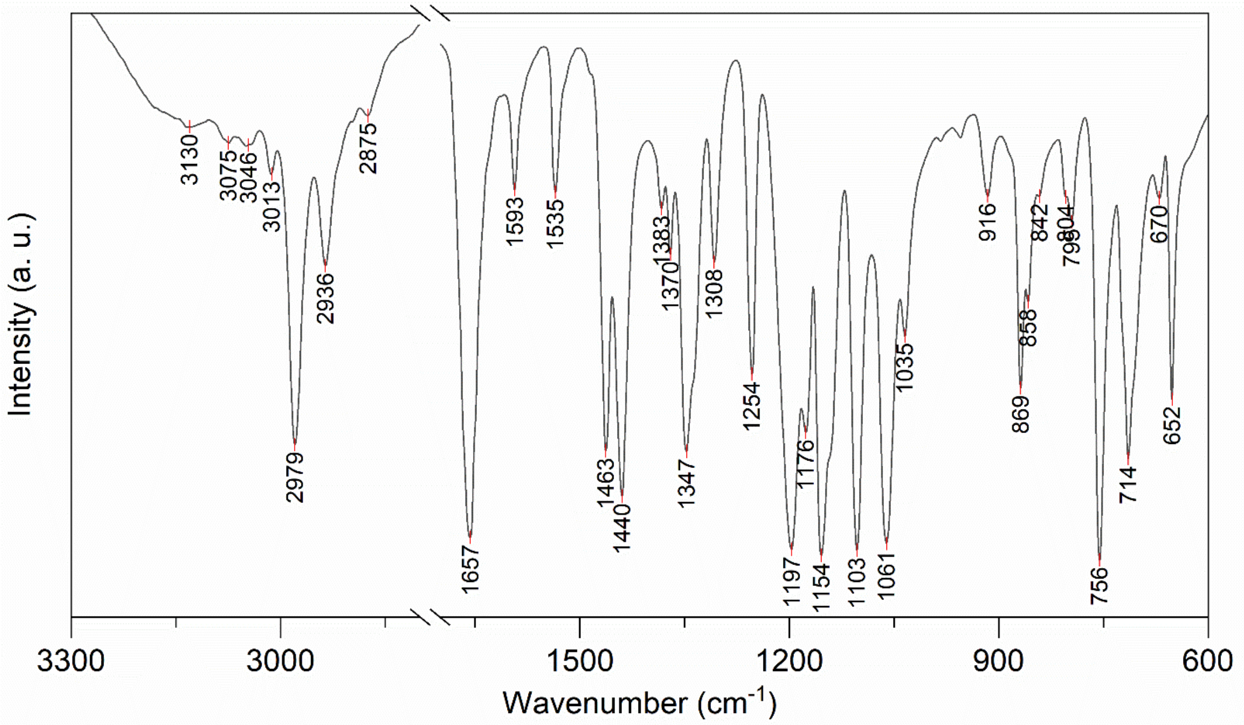

Figure S4. FT-IR spectrum of KL (IIc).

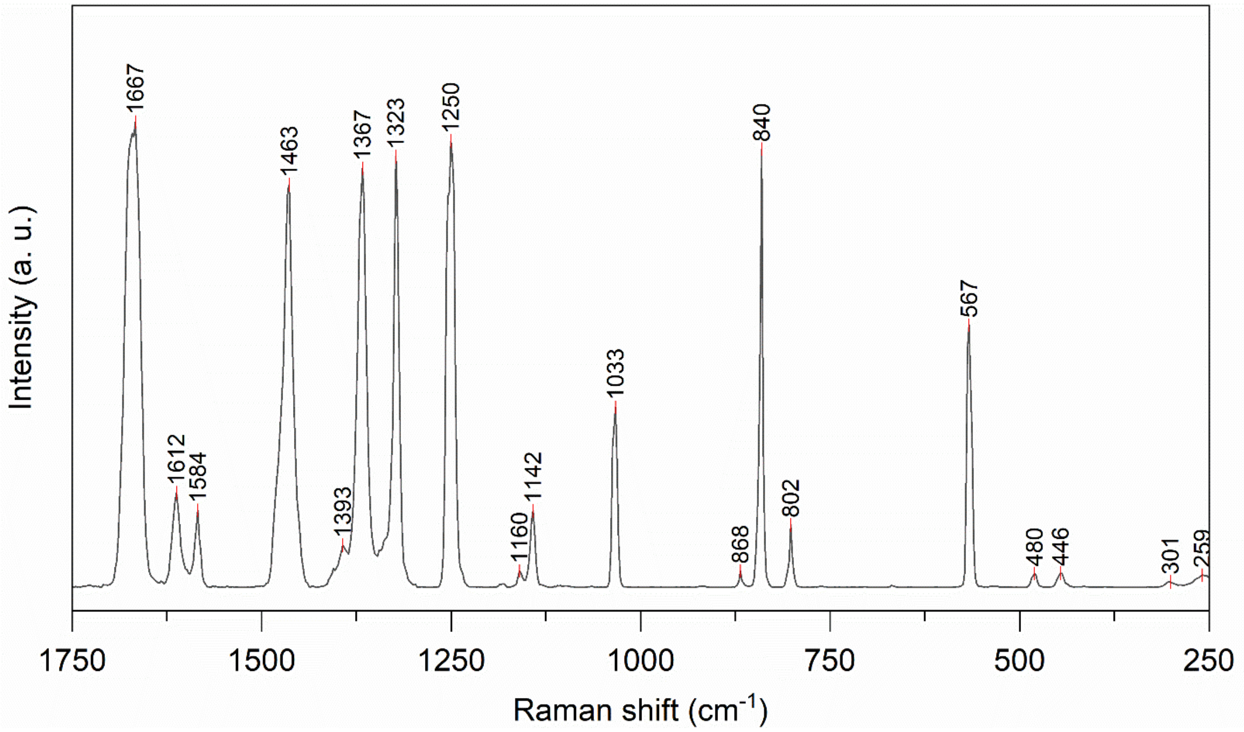

Figure S5. Raman spectrum of HL (II).

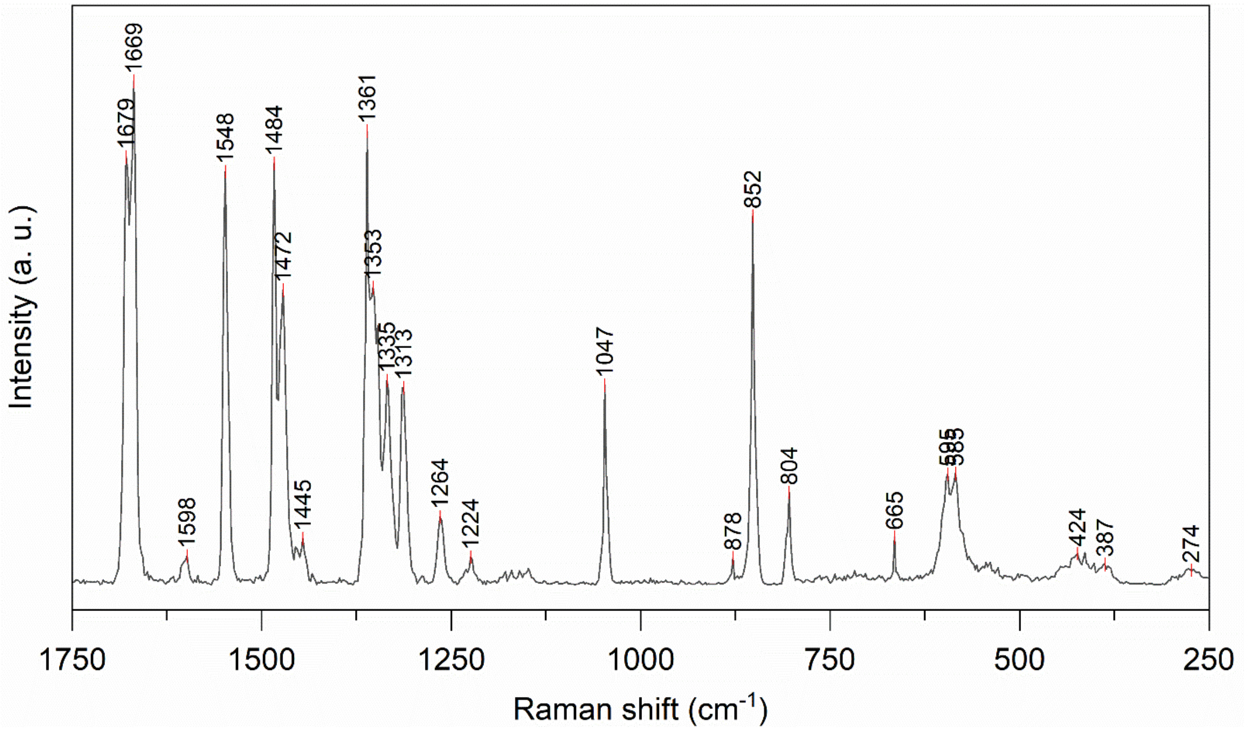

Figure S6. Raman spectrum of LiL (IIa).

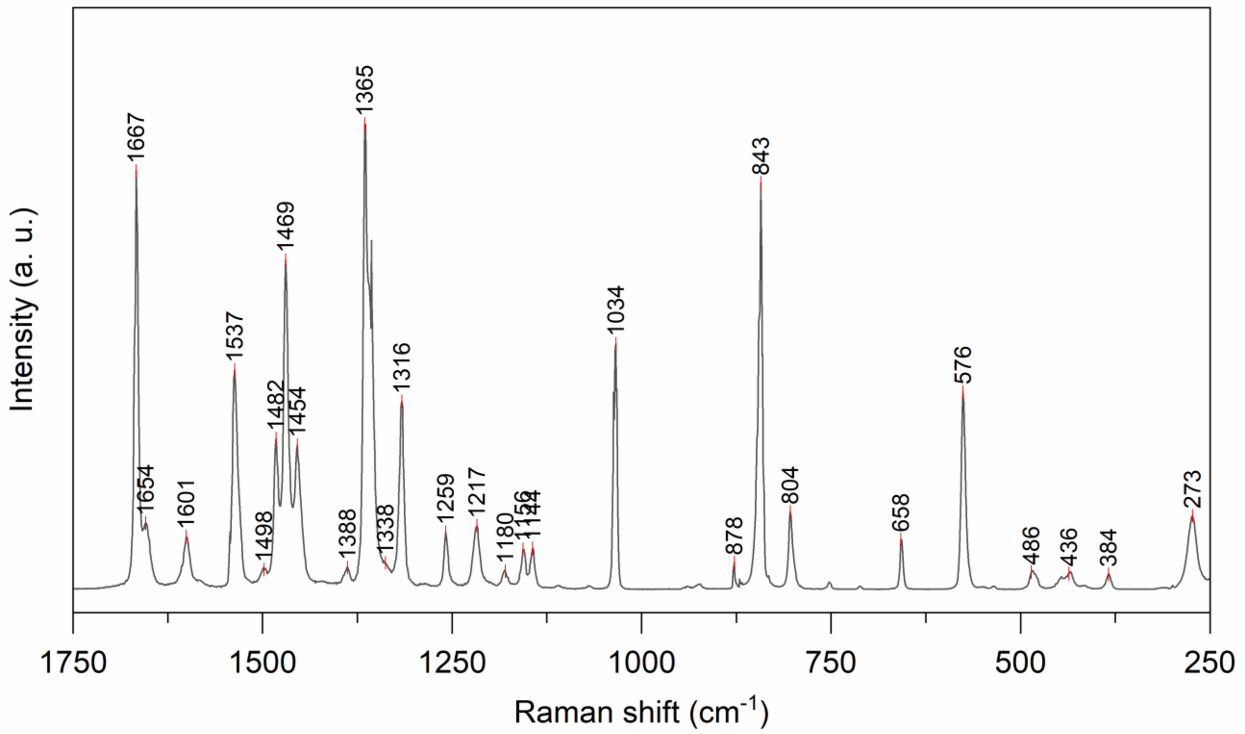

Figure S7. Raman spectrum of NaL (IIb).

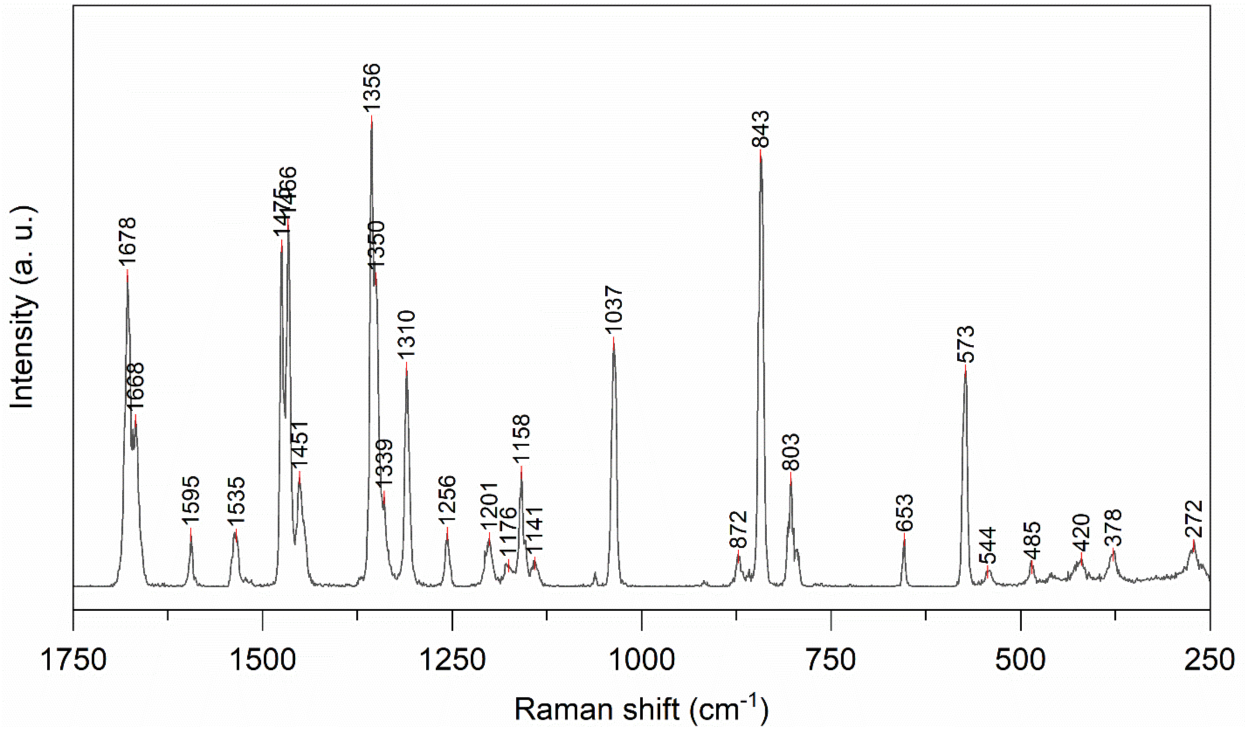

Figure S8. Raman spectrum of KL (IIc).

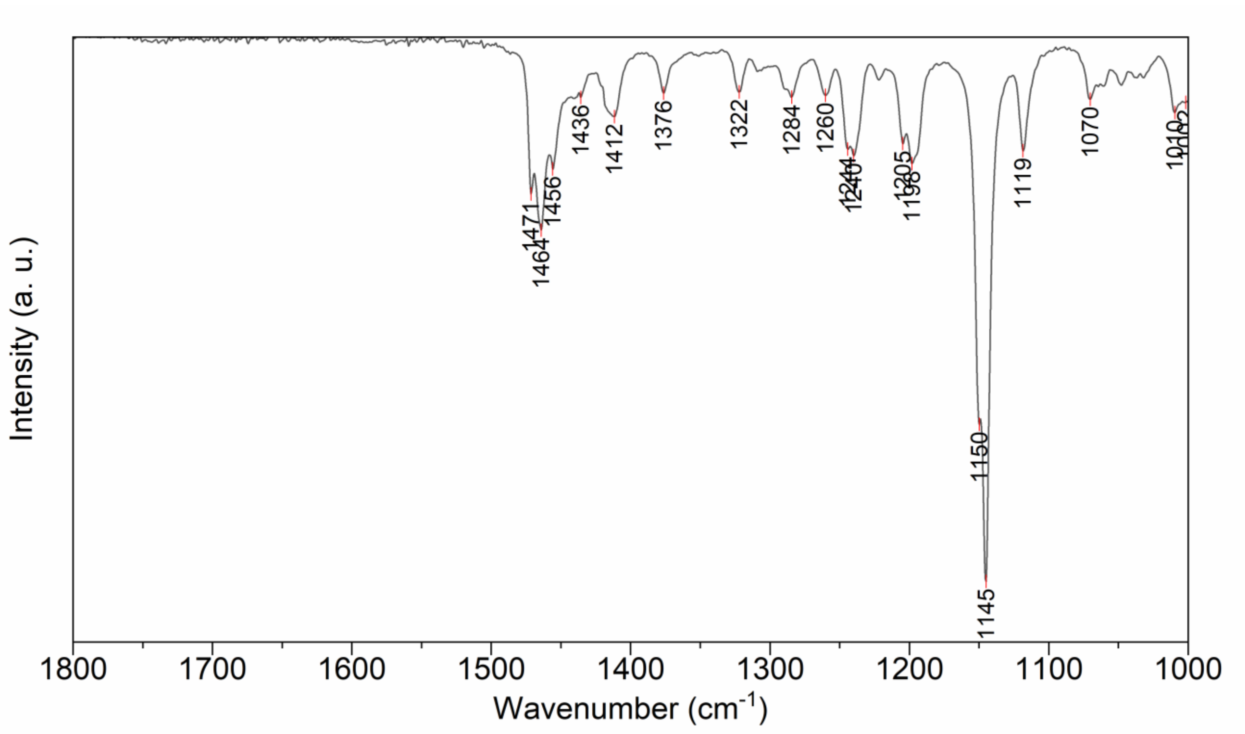

Figure S9. FT-IR spectrum of TOPO.

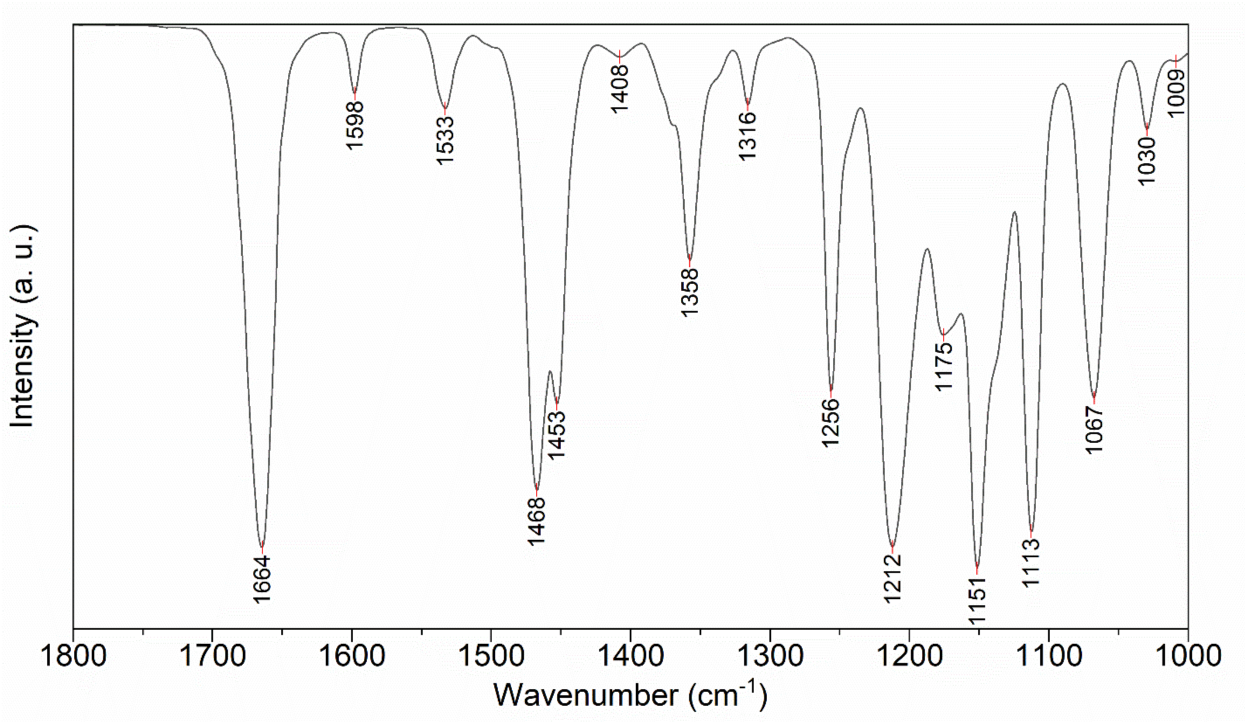

Figure S10. FT-IR spectrum of Li(L)TOPO (IIa).

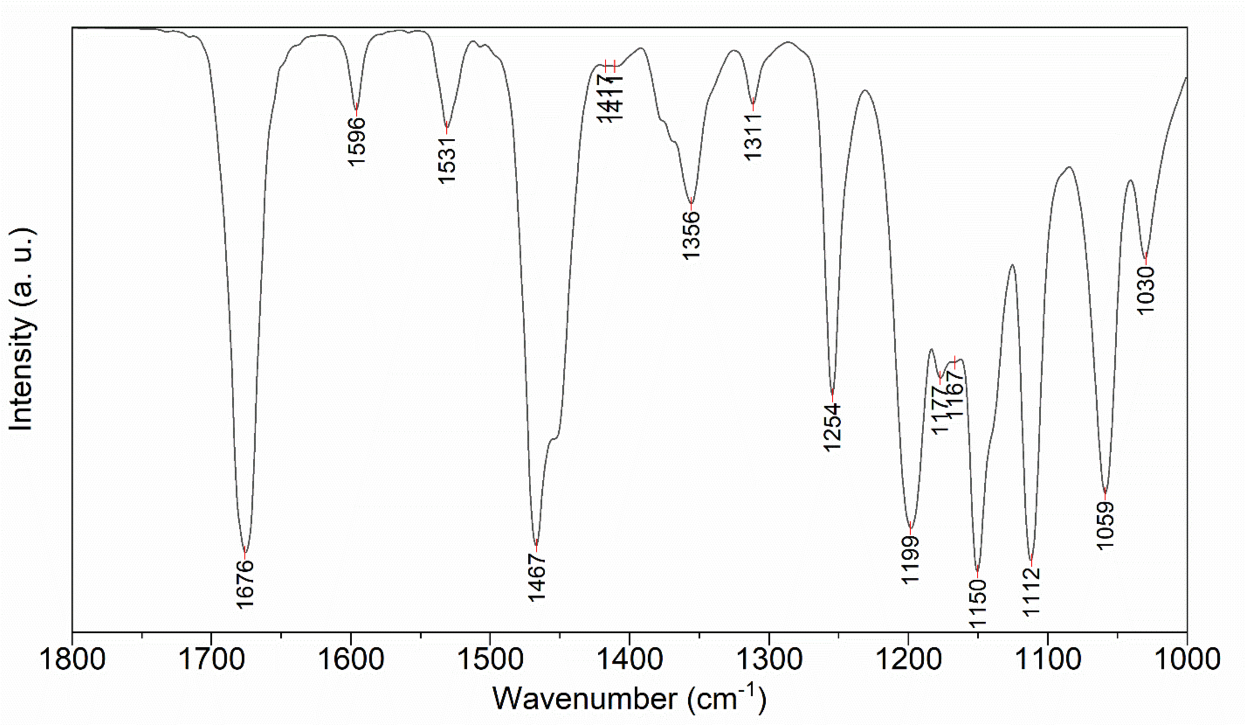

Figure S11. FT-IR spectrum of Na(L)TOPO (IIb).

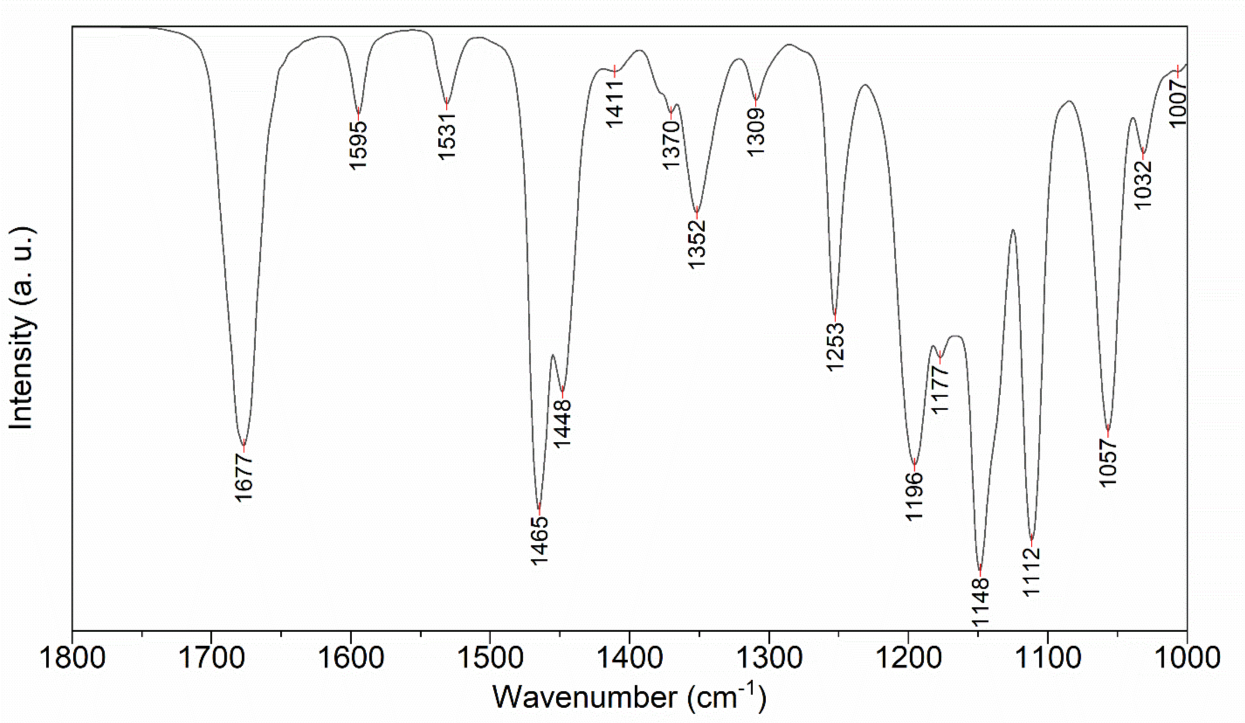

Figure S12. FT-IR spectrum of K(L)TOPO (IIc).

Table S4. Variation of 13P chemical shifts at different IPSAL-Li / TOPO ratio.

LiL / TOPO ratio	TOPO ratio. mol%	31P NMR			
		303K		223K	
		δ, ppm	Δδ, ppm	δ, ppm	Δδ, ppm
0:1	100	48.42	0	50.98	0
1:10	91	48.58	0.16	51.01	0.03
1:4	80	48.79	0.40	51.06	0.08
1:2	67	49.13	0.68	51.09	0.11
1:1.5	60	49.01	0.91	-	-
1:1.25	56	48.88	0.83	-	-
1:1	50	49.37	0.95	51.16	0.18
1:0.75	43	49.49	1.075	-	-
1:0.5	33	49.62	1.195	51.29	0.31

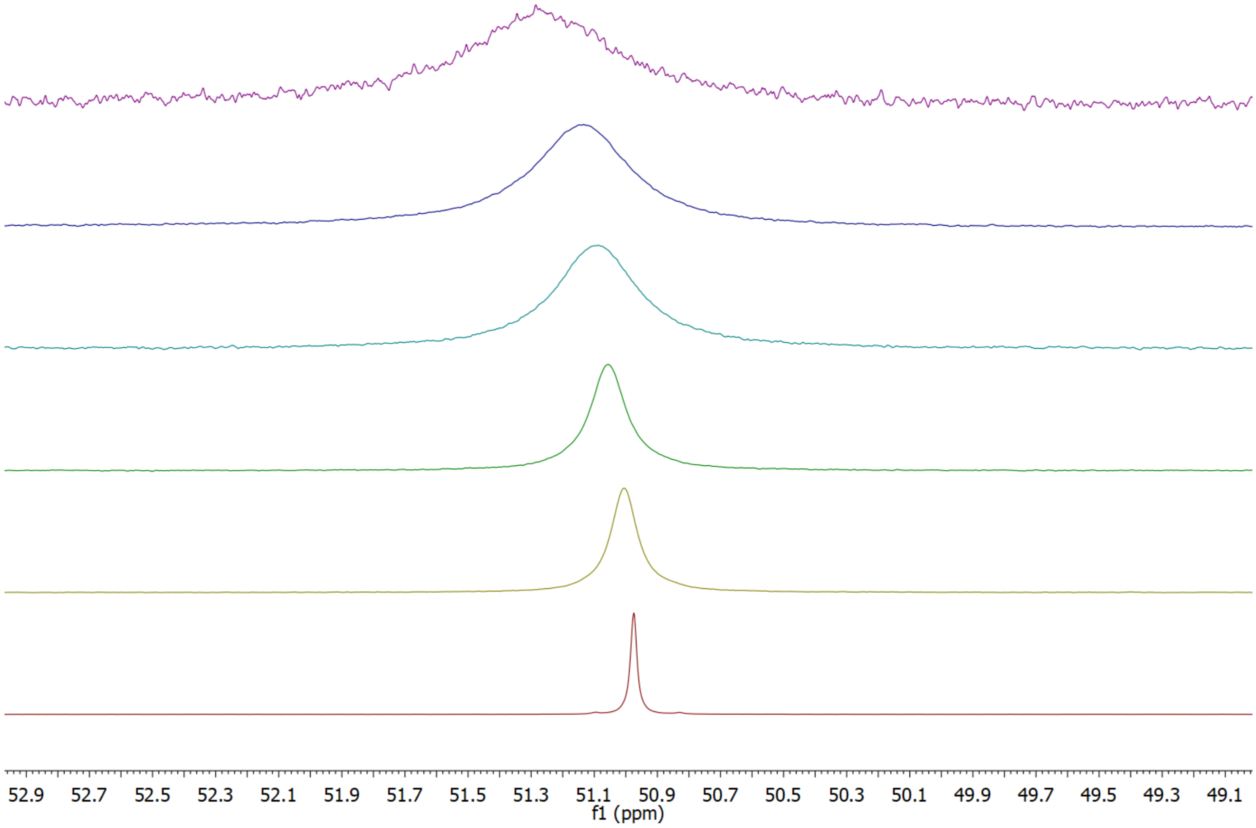

Figure S13. ^{31}P NMR spectra of the Li(L)TOPO system at various component ratio (303K, $CDCl_3$).

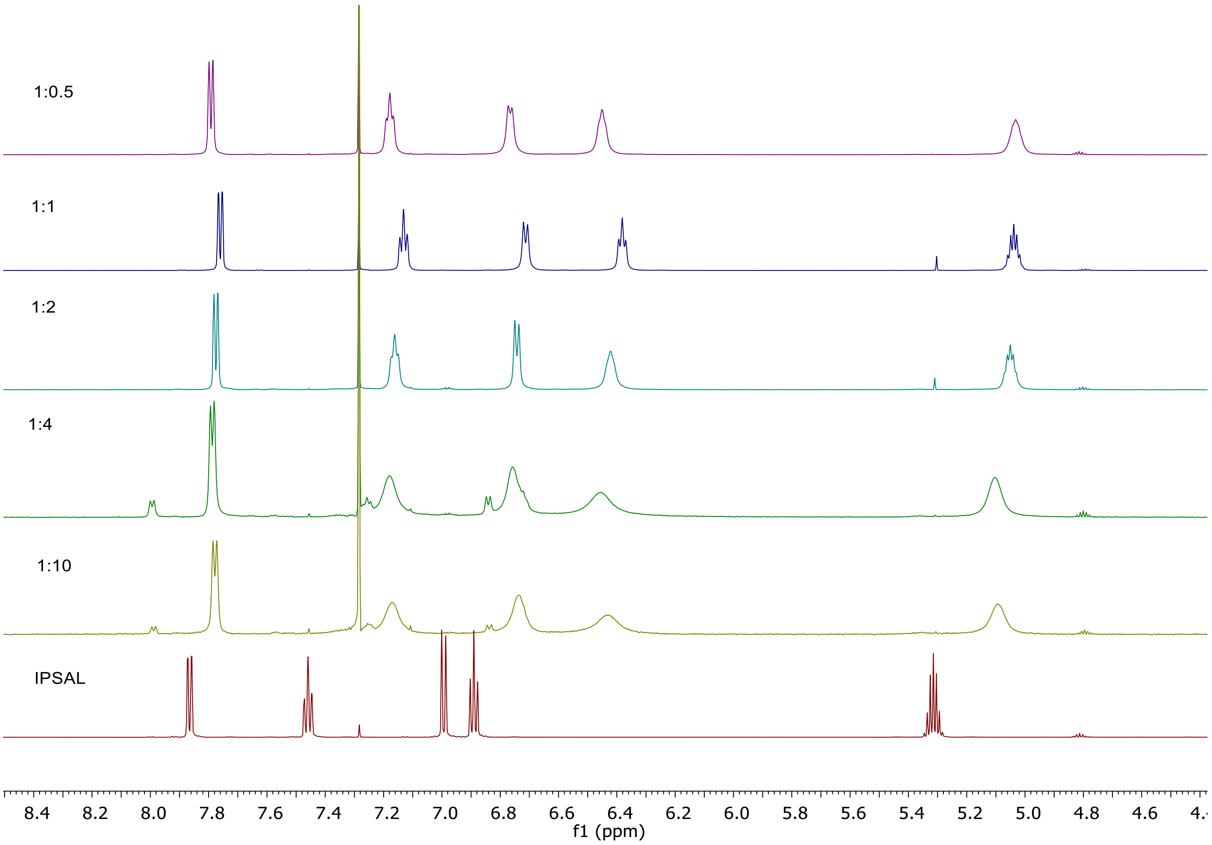

Figure S14. 1H NMR spectra of the Li(L)TOPO system at various component ratios (CDCl$_3$).

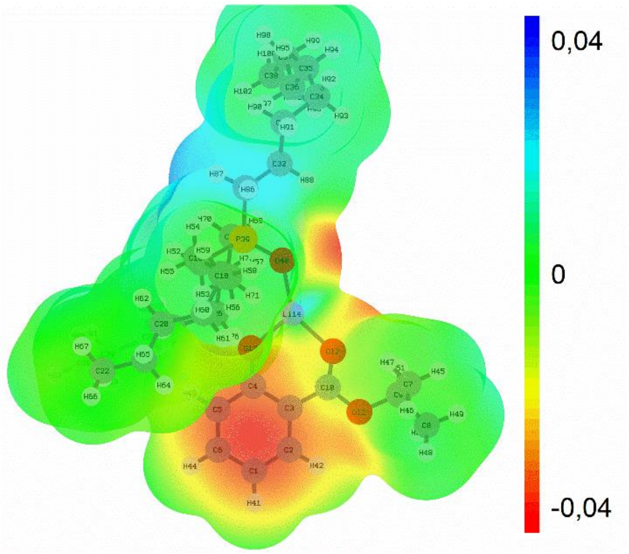

Figure S15. Electron density from the total SCF density (isoval = 0.0004) mapped with the electrostatic potential (ESP) for Li(L)TOPO.
